# Research ethics and artificial intelligence for global health: perspectives from the global forum on bioethics in research

**DOI:** 10.1186/s12910-024-01044-w

**Published:** 2024-04-18

**Authors:** James Shaw, Joseph Ali, Caesar A. Atuire, Phaik Yeong Cheah, Armando Guio Español, Judy Wawira Gichoya, Adrienne Hunt, Daudi Jjingo, Katherine Littler, Daniela Paolotti, Effy Vayena

**Affiliations:** 1 Department of Physical Therapy, Temerty Faculty of Medicine, University of Toronto, Toronto, Canada; 2 Berman Institute of Bioethics, Johns Hopkins University, Baltimore, MD USA; 3 Bloomberg School of Public Health, Johns Hopkins University, Baltimore, MD USA; 4 Department of Philosophy and Classics, University of Ghana, Legon-Accra, Ghana; 5 Centre for Tropical Medicine and Global Health, Nuffield Department of Medicine, University of Oxford, Oxford, UK; 6 Mahidol Oxford Tropical Medicine Research Unit, Faculty of Tropical Medicine, Mahidol University, Bangkok, Thailand; 7 Berkman Klein Center, Harvard University, Bogotá, Colombia; 8 Department of Radiology and Informatics, Emory University School of Medicine, Atlanta, GA USA; 9 Health Ethics & Governance Unit, Research for Health Department, Science Division, World Health Organization, Geneva, Switzerland; 10 African Center of Excellence in Bioinformatics and Data Intensive Science, Infectious Diseases Institute, Makerere University, Kampala, Uganda; 11 ISI Foundation, Turin, Italy; 12 Department of Health Sciences and Technology, ETH Zurich, Zürich, Switzerland; 13 Joint Centre for Bioethics, Dalla Lana School of Public Health, University of Toronto, Toronto, Canada

**Keywords:** Artificial intelligence, Machine learning, Bioethics, Research ethics, Global health

## Abstract

**Background:**

The ethical governance of Artificial Intelligence (AI) in health care and public health continues to be an urgent issue for attention in policy, research, and practice. In this paper we report on central themes related to challenges and strategies for promoting ethics in research involving AI in global health, arising from the Global Forum on Bioethics in Research (GFBR), held in Cape Town, South Africa in November 2022.

**Methods:**

The GFBR is an annual meeting organized by the World Health Organization and supported by the Wellcome Trust, the US National Institutes of Health, the UK Medical Research Council (MRC) and the South African MRC. The forum aims to bring together ethicists, researchers, policymakers, research ethics committee members and other actors to engage with challenges and opportunities specifically related to research ethics. In 2022 the focus of the GFBR was “Ethics of AI in Global Health Research”. The forum consisted of 6 case study presentations, 16 governance presentations, and a series of small group and large group discussions. A total of 87 participants attended the forum from 31 countries around the world, representing disciplines of bioethics, AI, health policy, health professional practice, research funding, and bioinformatics. In this paper, we highlight central insights arising from GFBR 2022.

**Results:**

We describe the significance of four thematic insights arising from the forum: (1) Appropriateness of building AI, (2) Transferability of AI systems, (3) Accountability for AI decision-making and outcomes, and (4) Individual consent. We then describe eight recommendations for governance leaders to enhance the ethical governance of AI in global health research, addressing issues such as AI impact assessments, environmental values, and fair partnerships.

**Conclusions:**

The 2022 Global Forum on Bioethics in Research illustrated several innovations in ethical governance of AI for global health research, as well as several areas in need of urgent attention internationally. This summary is intended to inform international and domestic efforts to strengthen research ethics and support the evolution of governance leadership to meet the demands of AI in global health research.

## Introduction

The ethical governance of Artificial Intelligence (AI) in health care and public health continues to be an urgent issue for attention in policy, research, and practice [1–3]. Beyond the growing number of AI applications being implemented in health care, capabilities of AI models such as Large Language Models (LLMs) expand the potential reach and significance of AI technologies across health-related fields [4, 5]. Discussion about effective, ethical governance of AI technologies has spanned a range of governance approaches, including government regulation, organizational decision-making, professional self-regulation, and research ethics review [6–8]. In this paper, we report on central themes related to challenges and strategies for promoting ethics in research involving AI in global health research, arising from the Global Forum on Bioethics in Research (GFBR), held in Cape Town, South Africa in November 2022. Although applications of AI for research, health care, and public health are diverse and advancing rapidly, the insights generated at the forum remain highly relevant from a global health perspective. After summarizing important context for work in this domain, we highlight categories of ethical issues emphasized at the forum for attention from a research ethics perspective internationally. We then outline strategies proposed for research, innovation, and governance to support more ethical AI for global health.

In this paper, we adopt the definition of AI systems provided by the Organization for Economic Cooperation and Development (OECD) as our starting point. Their definition states that an AI system is “a machine-based system that can, for a given set of human-defined objectives, make predictions, recommendations, or decisions influencing real or virtual environments. AI systems are designed to operate with varying levels of autonomy” [9]. The conceptualization of an algorithm as helping to constitute an AI system, along with hardware, other elements of software, and a particular context of use, illustrates the wide variety of ways in which AI can be applied. We have found it useful to differentiate applications of AI in research as those classified as “AI systems for discovery” and “AI systems for intervention”. An AI system for discovery is one that is intended to generate new knowledge, for example in drug discovery or public health research in which researchers are seeking potential targets for intervention, innovation, or further research. An AI system for intervention is one that directly contributes to enacting an intervention in a particular context, for example informing decision-making at the point of care or assisting with accuracy in a surgical procedure.

The mandate of the GFBR is to take a broad view of what constitutes research and its regulation in global health, with special attention to bioethics in Low- and Middle- Income Countries. AI as a group of technologies demands such a broad view. AI development for health occurs in a variety of environments, including universities and academic health sciences centers where research ethics review remains an important element of the governance of science and innovation internationally [10, 11]. In these settings, research ethics committees (RECs; also known by different names such as Institutional Review Boards or IRBs) make decisions about the ethical appropriateness of projects proposed by researchers and other institutional members, ultimately determining whether a given project is allowed to proceed on ethical grounds [12].

However, research involving AI for health also takes place in large corporations and smaller scale start-ups, which in some jurisdictions fall outside the scope of research ethics regulation. In the domain of AI, the question of what constitutes research also becomes blurred. For example, is the development of an algorithm itself considered a part of the research process? Or only when that algorithm is tested under the formal constraints of a systematic research methodology? In this paper we take an inclusive view, in which AI development is included in the definition of research activity and within scope for our inquiry, regardless of the setting in which it takes place. This broad perspective characterizes the approach to “research ethics” we take in this paper, extending beyond the work of RECs to include the ethical analysis of the wide range of activities that constitute research as the generation of new knowledge and intervention in the world.

### Ethical governance of AI in global health

The ethical governance of AI for global health has been widely discussed in recent years. The World Health Organization (WHO) released its guidelines on ethics and governance of AI for health in 2021, endorsing a set of six ethical principles and exploring the relevance of those principles through a variety of use cases. The WHO guidelines also provided an overview of AI governance, defining governance as covering “a range of steering and rule-making functions of governments and other decision-makers, including international health agencies, for the achievement of national health policy objectives conducive to universal health coverage.” (p. 81) The report usefully provided a series of recommendations related to governance of seven domains pertaining to AI for health: data, benefit sharing, the private sector, the public sector, regulation, policy observatories/model legislation, and global governance. The report acknowledges that much work is yet to be done to advance international cooperation on AI governance, especially related to prioritizing voices from Low- and Middle-Income Countries (LMICs) in global dialogue.

One important point emphasized in the WHO report that reinforces the broader literature on global governance of AI is the distribution of responsibility across a wide range of actors in the AI ecosystem. This is especially important to highlight when focused on research for global health, which is specifically about work that transcends national borders. Alami et al. (2020) discussed the unique risks raised by AI research in global health, ranging from the unavailability of data in many LMICs required to train locally relevant AI models to the capacity of health systems to absorb new AI technologies that demand the use of resources from elsewhere in the system. These observations illustrate the need to identify the unique issues posed by AI research for global health specifically, and the strategies that can be employed by all those implicated in AI governance to promote ethically responsible use of AI in global health research.

### RECs and the regulation of research involving AI

RECs represent an important element of the governance of AI for global health research, and thus warrant further commentary as background to our paper. Despite the importance of RECs, foundational questions have been raised about their capabilities to accurately understand and address ethical issues raised by studies involving AI. Rahimzadeh et al. (2023) outlined how RECs in the United States are under-prepared to align with recent federal policy requiring that RECs review data sharing and management plans with attention to the unique ethical issues raised in AI research for health [13]. Similar research in South Africa identified variability in understanding of existing regulations and ethical issues associated with health-related big data sharing and management among research ethics committee members [14, 15]. The effort to address harms accruing to groups or communities as opposed to individuals whose data are included in AI research has also been identified as a unique challenge for RECs [16, 17]. Doerr and Meeder (2022) suggested that current regulatory frameworks for research ethics might actually prevent RECs from adequately addressing such issues, as they are deemed out of scope of REC review [16]. Furthermore, research in the United Kingdom and Canada has suggested that researchers using AI methods for health tend to distinguish between ethical issues and social impact of their research, adopting an overly narrow view of what constitutes ethical issues in their work [18].

The challenges for RECs in adequately addressing ethical issues in AI research for health care and public health exceed a straightforward survey of ethical considerations. As Ferretti et al. (2021) contend, some capabilities of RECs adequately cover certain issues in AI-based health research, such as the common occurrence of conflicts of interest where researchers who accept funds from commercial technology providers are implicitly incentivized to produce results that align with commercial interests [12]. However, some features of REC review require reform to adequately meet ethical needs. Ferretti et al. outlined weaknesses of RECs that are longstanding and those that are novel to AI-related projects, proposing a series of directions for development that are regulatory, procedural, and complementary to REC functionality. The work required on a global scale to update the REC function in response to the demands of research involving AI is substantial.

These issues take greater urgency in the context of global health [19]. Teixeira da Silva (2022) described the global practice of “ethics dumping”, where researchers from high income countries bring ethically contentious practices to RECs in low-income countries as a strategy to gain approval and move projects forward [20]. Although not yet systematically documented in AI research for health, risk of ethics dumping in AI research is high. Evidence is already emerging of practices of “health data colonialism”, in which AI researchers and developers from large organizations in high-income countries acquire data to build algorithms in LMICs to avoid stricter regulations [21]. This specific practice is part of a larger collection of practices that characterize health data colonialism, involving the broader exploitation of data and the populations they represent primarily for commercial gain [21, 22]. As an additional complication, AI algorithms trained on data from high-income contexts are unlikely to apply in straightforward ways to LMIC settings [21, 23]. In the context of global health, there is widespread acknowledgement about the need to not only enhance the knowledge base of REC members about AI-based methods internationally, but to acknowledge the broader shifts required to encourage their capabilities to more fully address these and other ethical issues associated with AI research for health [8].

Although RECs are an important part of the story of the ethical governance of AI for global health research, they are not the only part. The responsibilities of supra-national entities such as the World Health Organization, national governments, organizational leaders, commercial AI technology providers, health care professionals, and other groups continue to be worked out internationally. In this context of ongoing work, examining issues that demand attention and strategies to address them remains an urgent and valuable task.

## Methods

The GFBR is an annual meeting organized by the World Health Organization and supported by the Wellcome Trust, the US National Institutes of Health, the UK Medical Research Council (MRC) and the South African MRC. The forum aims to bring together ethicists, researchers, policymakers, REC members and other actors to engage with challenges and opportunities specifically related to research ethics. Each year the GFBR meeting includes a series of case studies and keynotes presented in plenary format to an audience of approximately 100 people who have applied and been competitively selected to attend, along with small-group breakout discussions to advance thinking on related issues. The specific topic of the forum changes each year, with past topics including ethical issues in research with people living with mental health conditions (2021), genome editing (2019), and biobanking/data sharing (2018). The forum is intended to remain grounded in the practical challenges of engaging in research ethics, with special interest in low resource settings from a global health perspective. A post-meeting fellowship scheme is open to all LMIC participants, providing a unique opportunity to apply for funding to further explore and address the ethical challenges that are identified during the meeting.

In 2022, the focus of the GFBR was “Ethics of AI in Global Health Research”. The forum consisted of 6 case study presentations (both short and long form) reporting on specific initiatives related to research ethics and AI for health, and 16 governance presentations (both short and long form) reporting on actual approaches to governing AI in different country settings. A keynote presentation from Professor Effy Vayena addressed the topic of the broader context for AI ethics in a rapidly evolving field. A total of 87 participants attended the forum from 31 countries around the world, representing disciplines of bioethics, AI, health policy, health professional practice, research funding, and bioinformatics. The 2-day forum addressed a wide range of themes. The conference report provides a detailed overview of each of the specific topics addressed while a policy paper outlines the cross-cutting themes (both documents are available at the GFBR website: https://www.gfbr.global/past-meetings/16th-forum-cape-town-south-africa-29-30-november-2022/). As opposed to providing a detailed summary in this paper, we aim to briefly highlight central issues raised, solutions proposed, and the challenges facing the research ethics community in the years to come.

In this way, our primary aim in this paper is to present a synthesis of the challenges and opportunities raised at the GFBR meeting and in the planning process, followed by our reflections as a group of authors on their significance for governance leaders in the coming years. We acknowledge that the views represented at the meeting and in our results are a partial representation of the universe of views on this topic; however, the GFBR leadership invested a great deal of resources in convening a deeply diverse and thoughtful group of researchers and practitioners working on themes of bioethics related to AI for global health including those based in LMICs. We contend that it remains rare to convene such a strong group for an extended time and believe that many of the challenges and opportunities raised demand attention for more ethical futures of AI for health. Nonetheless, our results are primarily descriptive and are thus not explicitly grounded in a normative argument. We make effort in the Discussion section to contextualize our results by describing their significance and connecting them to broader efforts to reform global health research and practice.

## Results

### Uniquely important ethical issues for AI in global health research

Presentations and group dialogue over the course of the forum raised several issues for consideration, and here we describe four overarching themes for the ethical governance of AI in global health research. Brief descriptions of each issue can be found in Table 1. Reports referred to throughout the paper are available at the GFBR website provided above.

The first overarching thematic issue relates to the appropriateness of building AI technologies in response to health-related challenges in the first place. Case study presentations referred to initiatives where AI technologies were highly appropriate, such as in ear shape biometric identification to more accurately link electronic health care records to individual patients in Zambia (Alinani Simukanga). Although important ethical issues were raised with respect to privacy, trust, and community engagement in this initiative, the AI-based solution was appropriately matched to the challenge of accurately linking electronic records to specific patient identities. In contrast, forum participants raised questions about the appropriateness of an initiative using AI to improve the quality of handwashing practices in an acute care hospital in India (Niyoshi Shah), which led to gaming the algorithm. Overall, participants acknowledged the dangers of techno-solutionism, in which AI researchers and developers treat AI technologies as the most obvious solutions to problems that in actuality demand much more complex strategies to address [24]. However, forum participants agreed that RECs in different contexts have differing degrees of power to raise issues of the appropriateness of an AI-based intervention.

The second overarching thematic issue related to whether and how AI-based systems transfer from one national health context to another. One central issue raised by a number of case study presentations related to the challenges of validating an algorithm with data collected in a local environment. For example, one case study presentation described a project that would involve the collection of personally identifiable data for sensitive group identities, such as tribe, clan, or religion, in the jurisdictions involved (South Africa, Nigeria, Tanzania, Uganda and the US; Gakii Masunga). Doing so would enable the team to ensure that those groups were adequately represented in the dataset to ensure the resulting algorithm was not biased against specific community groups when deployed in that context. However, some members of these communities might desire to be represented in the dataset, whereas others might not, illustrating the need to balance autonomy and inclusivity. It was also widely recognized that collecting these data is an immense challenge, particularly when historically oppressive practices have led to a low-trust environment for international organizations and the technologies they produce. It is important to note that in some countries such as South Africa and Rwanda, it is illegal to collect information such as race and tribal identities, re-emphasizing the importance for cultural awareness and avoiding “one size fits all” solutions.

The third overarching thematic issue is related to understanding accountabilities for both the impacts of AI technologies and governance decision-making regarding their use. Where global health research involving AI leads to longer-term harms that might fall outside the usual scope of issues considered by a REC, who is to be held accountable, and how? This question was raised as one that requires much further attention, with law being mixed internationally regarding the mechanisms available to hold researchers, innovators, and their institutions accountable over the longer term. However, it was recognized in breakout group discussion that many jurisdictions are developing strong data protection regimes related specifically to international collaboration for research involving health data. For example, Kenya’s Data Protection Act requires that any internationally funded projects have a local principal investigator who will hold accountability for how data are shared and used [25]. The issue of research partnerships with commercial entities was raised by many participants in the context of accountability, pointing toward the urgent need for clear principles related to strategies for engagement with commercial technology companies in global health research.

The fourth and final overarching thematic issue raised here is that of consent. The issue of consent was framed by the widely shared recognition that models of individual, explicit consent might not produce a supportive environment for AI innovation that relies on the secondary uses of health-related datasets to build AI algorithms. Given this recognition, approaches such as community oversight of health data uses were suggested as a potential solution. However, the details of implementing such community oversight mechanisms require much further attention, particularly given the unique perspectives on health data in different country settings in global health research. Furthermore, some uses of health data do continue to require consent. One case study of South Africa, Nigeria, Kenya, Ethiopia and Uganda suggested that when health data are shared across borders, individual consent remains necessary when data is transferred from certain countries (Nezerith Cengiz). Broader clarity is necessary to support the ethical governance of health data uses for AI in global health research.

**Table 1:** Overarching thematic issues

Issue	Brief Description
Appropriateness of building AI	A central question that must be asked when exploring the ethical status of AI-focused projects is whether it is appropriate or necessary to build AI in the first place
Transferability of AI systems	Whether and how AI systems can be transferred and function in new jurisdictional contexts or communities, and what new training processes might be required to ensure their benefit
Accountability for AI decision-making and outcomes	How accountabilities are identified in the development and deployment of AI systems where many actors are involved and often located in different jurisdictions. What guidelines apply for accountabilities in partnership with commercial entities
Individual Consent	Conventional notions of consent have been compromised by the secondary use of ever-larger datasets to develop AI technologies. When and how data from publics are used in such projects requires new models of considering consent, which is a central issue from a research ethics perspective

### Recommendations for ethical governance of AI in global health research

Dialogue at the forum led to a range of suggestions for promoting ethical conduct of AI research for global health, related to the various roles of actors involved in the governance of AI research broadly defined. The strategies are written for actors we refer to as “governance leaders”, those people distributed throughout the AI for global health research ecosystem who are responsible for ensuring the ethical and socially responsible conduct of global health research involving AI (including researchers themselves). These include RECs, government regulators, health care leaders, health professionals, corporate social accountability officers, and others. Enacting these strategies would bolster the ethical governance of AI for global health more generally, enabling multiple actors to fulfill their roles related to governing research and development activities carried out across multiple organizations, including universities, academic health sciences centers, start-ups, and technology corporations. Specific suggestions are summarized in Table 2.

First, forum participants suggested that governance leaders including RECs, should remain up to date on recent advances in the regulation of AI for health. Regulation of AI for health advances rapidly and takes on different forms in jurisdictions around the world. RECs play an important role in governance, but only a partial role; it was deemed important for RECs to acknowledge how they fit within a broader governance ecosystem in order to more effectively address the issues within their scope. Not only RECs but organizational leaders responsible for procurement, researchers, and commercial actors should all commit to efforts to remain up to date about the relevant approaches to regulating AI for health care and public health in jurisdictions internationally. In this way, governance can more adequately remain up to date with advances in regulation.

Second, forum participants suggested that governance leaders should focus on ethical governance of health data as a basis for ethical global health AI research. Health data are considered the foundation of AI development, being used to train AI algorithms for various uses [26]. By focusing on ethical governance of health data generation, sharing, and use, multiple actors will help to build an ethical foundation for AI development among global health researchers.

Third, forum participants believed that governance processes should incorporate AI impact assessments where appropriate. An AI impact assessment is the process of evaluating the potential effects, both positive and negative, of implementing an AI algorithm on individuals, society, and various stakeholders, generally over time frames specified in advance of implementation [27]. Although not all types of AI research in global health would warrant an AI impact assessment, this is especially relevant for those studies aiming to implement an AI system for intervention into health care or public health. Organizations such as RECs can use AI impact assessments to boost understanding of potential harms at the outset of a research project, encouraging researchers to more deeply consider potential harms in the development of their study.

Fourth, forum participants suggested that governance decisions should incorporate the use of environmental impact assessments, or at least the incorporation of environment values when assessing the potential impact of an AI system. An environmental impact assessment involves evaluating and anticipating the potential environmental effects of a proposed project to inform ethical decision-making that supports sustainability [28]. Although a relatively new consideration in research ethics conversations [29], the environmental impact of building technologies is a crucial consideration for the public health commitment to environmental sustainability. Governance leaders can use environmental impact assessments to boost understanding of potential environmental harms linked to AI research projects in global health over both the shorter and longer terms.

Fifth, forum participants suggested that governance leaders should require stronger transparency in the development of AI algorithms in global health research. Transparency was considered essential in the design and development of AI algorithms for global health to ensure ethical and accountable decision-making throughout the process. Furthermore, whether and how researchers have considered the unique contexts into which such algorithms may be deployed can be surfaced through stronger transparency, for example in describing what primary considerations were made at the outset of the project and which stakeholders were consulted along the way. Sharing information about data provenance and methods used in AI development will also enhance the trustworthiness of the AI-based research process.

Sixth, forum participants suggested that governance leaders can encourage or require community engagement at various points throughout an AI project. It was considered that engaging patients and communities is crucial in AI algorithm development to ensure that the technology aligns with community needs and values. However, participants acknowledged that this is not a straightforward process. Effective community engagement requires lengthy commitments to meeting with and hearing from diverse communities in a given setting, and demands a particular set of skills in communication and dialogue that are not possessed by all researchers. Encouraging AI researchers to begin this process early and build long-term partnerships with community members is a promising strategy to deepen community engagement in AI research for global health. One notable recommendation was that research funders have an opportunity to incentivize and enable community engagement with funds dedicated to these activities in AI research in global health.

Seventh, forum participants suggested that governance leaders can encourage researchers to build strong, fair partnerships between institutions and individuals across country settings. In a context of longstanding imbalances in geopolitical and economic power, fair partnerships in global health demand a priori commitments to share benefits related to advances in medical technologies, knowledge, and financial gains. Although enforcement of this point might be beyond the remit of RECs, commentary will encourage researchers to consider stronger, fairer partnerships in global health in the longer term.

Eighth, it became evident that it is necessary to explore new forms of regulatory experimentation given the complexity of regulating a technology of this nature. In addition, the health sector has a series of particularities that make it especially complicated to generate rules that have not been previously tested. Several participants highlighted the desire to promote spaces for experimentation such as regulatory sandboxes or innovation hubs in health. These spaces can have several benefits for addressing issues surrounding the regulation of AI in the health sector, such as: (i) increasing the capacities and knowledge of health authorities about this technology; (ii) identifying the major problems surrounding AI regulation in the health sector; (iii) establishing possibilities for exchange and learning with other authorities; (iv) promoting innovation and entrepreneurship in AI in health; and (vi) identifying the need to regulate AI in this sector and update other existing regulations.

Ninth and finally, forum participants believed that the capabilities of governance leaders need to evolve to better incorporate expertise related to AI in ways that make sense within a given jurisdiction. With respect to RECs, for example, it might not make sense for every REC to recruit a member with expertise in AI methods. Rather, it will make more sense in some jurisdictions to consult with members of the scientific community with expertise in AI when research protocols are submitted that demand such expertise. Furthermore, RECs and other approaches to research governance in jurisdictions around the world will need to evolve in order to adopt the suggestions outlined above, developing processes that apply specifically to the ethical governance of research using AI methods in global health.

**Table 2:** Suggestions for governance leaders related to AI for global health research

Strategies to Promote Ethical AI for Global Health	Brief Description
Remain up to date on recent advances in the regulation of AI for health	Regulation of AI for health advances rapidly and takes on different forms in jurisdictions around the world. Governance leaders should commit to efforts to remain up to date about the relevant approaches to regulating AI for health care and public health in the jurisdictions internationally
Focus on ethical governance of health data	Health data are the foundation of AI development. Emphasis on ethical governance of health data generation, sharing, and use will help to build an ethical foundation for AI development
Incorporate AI impact assessments	An AI impact assessment is the process of evaluating the potential effects, both positive and negative, of implementing an AI algorithm on individuals, society, and various stakeholders, generally over time frames specified in advance of implementation. Governance leaders can use AI impact assessments to boost understanding of potential harms
Incorporate environmental impact assessments	An environmental impact assessment involves evaluating and predicting the potential environmental effects of a proposed project to inform ethical decision-making that supports sustainability. Governance leaders can use environmental impact assessments to boost understanding of potential environmental harms
Promote transparency in AI development	Governance leaders can request information to promote transparency in the AI development process. Transparency is crucial in developing AI algorithms for health care to ensure ethical and accountable decision-making
Encourage community engagement across phases of AI development	Governance leaders can encourage or require community engagement at various points throughout an AI project. Engaging patients and communities is important in AI algorithm development as it ensures that the technology aligns with community needs and values
Encourage fair partnership between researchers and organizations in different jurisdictions involved	Governance leaders can encourage researchers to build AI development for global health on a foundation of fair partnerships between institutions and individuals across countries, with the aims of enabling equitable benefit through access to advanced medical technologies, knowledge sharing, and sharing of financial benefits
Explore new forms of regulatory experimentation for AI	New approaches to regulating AI technologies for health can be explored in experimental spaces that allow for a deeper understanding of their benefits and drawbacks
Evolve the structure and capabilities of governance functions	The capabilities of governance functions and individual governance leaders need to evolve to better incorporate expertise related to AI in ways that make sense within a given jurisdiction

## Discussion

Research involving the development and implementation of AI technologies continues to grow in global health, posing important challenges for ethical governance of AI in global health research around the world. In this paper we have summarized insights from the 2022 GFBR, focused specifically on issues in research ethics related to AI for global health research. We summarized four thematic challenges for governance related to AI in global health research and nine suggestions arising from presentations and dialogue at the forum. In this brief discussion section, we present an overarching observation about power imbalances that frames efforts to evolve the role of governance in global health research, and then outline two important opportunity areas as the field develops to meet the challenges of AI in global health research.

Dialogue about power is not unfamiliar in global health, especially given recent contributions exploring what it would mean to de-colonize global health research, funding, and practice [30, 31]. Discussions of research ethics applied to AI research in global health contexts are deeply infused with power imbalances. The existing context of global health is one in which high-income countries primarily located in the “Global North” charitably invest in projects taking place primarily in the “Global South” while recouping knowledge, financial, and reputational benefits [32]. With respect to AI development in particular, recent examples of digital colonialism frame dialogue about global partnerships, raising attention to the role of large commercial entities and global financial capitalism in global health research [21, 22]. Furthermore, the power of governance organizations such as RECs to intervene in the process of AI research in global health varies widely around the world, depending on the authorities assigned to them by domestic research governance policies. These observations frame the challenges outlined in our paper, highlighting the difficulties associated with making meaningful change in this field.

Despite these overarching challenges of the global health research context, there are clear strategies for progress in this domain. Firstly, AI innovation is rapidly evolving, which means approaches to the governance of AI for health are rapidly evolving too. Such rapid evolution presents an important opportunity for governance leaders to clarify their vision and influence over AI innovation in global health research, boosting the expertise, structure, and functionality required to meet the demands of research involving AI. Secondly, the research ethics community has strong international ties, linked to a global scholarly community that is committed to sharing insights and best practices around the world. This global community can be leveraged to coordinate efforts to produce advances in the capabilities and authorities of governance leaders to meaningfully govern AI research for global health given the challenges summarized in our paper.

### Limitations

Our paper includes two specific limitations that we address explicitly here. First, it is still early in the lifetime of the development of applications of AI for use in global health, and as such, the global community has had limited opportunity to learn from experience. For example, there were many fewer case studies, which detail experiences with the actual implementation of an AI technology, submitted to GFBR 2022 for consideration than was expected. In contrast, there were many more governance reports submitted, which detail the processes and outputs of governance processes that anticipate the development and dissemination of AI technologies. This observation represents both a success and a challenge. It is a success that so many groups are engaging in anticipatory governance of AI technologies, exploring evidence of their likely impacts and governing technologies in novel and well-designed ways. It is a challenge that there is little experience to build upon of the successful implementation of AI technologies in ways that have limited harms while promoting innovation. Further experience with AI technologies in global health will contribute to revising and enhancing the challenges and recommendations we have outlined in our paper.

Second, global trends in the politics and economics of AI technologies are evolving rapidly. Although some nations are advancing detailed policy approaches to regulating AI more generally, including for uses in health care and public health, the impacts of corporate investments in AI and political responses related to governance remain to be seen. The excitement around large language models (LLMs) and large multimodal models (LMMs) has drawn deeper attention to the challenges of regulating AI in any general sense, opening dialogue about health sector-specific regulations. The direction of this global dialogue, strongly linked to high-profile corporate actors and multi-national governance institutions, will strongly influence the development of boundaries around what is possible for the ethical governance of AI for global health. We have written this paper at a point when these developments are proceeding rapidly, and as such, we acknowledge that our recommendations will need updating as the broader field evolves.

## Conclusion

Ultimately, coordination and collaboration between many stakeholders in the research ethics ecosystem will be necessary to strengthen the ethical governance of AI in global health research. The 2022 GFBR illustrated several innovations in ethical governance of AI for global health research, as well as several areas in need of urgent attention internationally. This summary is intended to inform international and domestic efforts to strengthen research ethics and support the evolution of governance leadership to meet the demands of AI in global health research.

## Data Availability

All data and materials analyzed to produce this paper are available on the GFBR website: https://www.gfbr.global/past-meetings/16th-forum-cape-town-south-africa-29-30-november-2022/.
